# Relevant Properties of Carbon Support Materials in Successful Fe-N-C Synthesis for the Oxygen Reduction Reaction: Study of Carbon Blacks and Biomass-Based Carbons

**DOI:** 10.3390/ma14010045

**Published:** 2020-12-24

**Authors:** Julia Hülstede, Dana Schonvogel, Henrike Schmies, Peter Wagner, Frank Schröter, Alexander Dyck, Michael Wark

**Affiliations:** 1 German Aerospace Center (DLR), Institute of Networked Energy Systems, 26129 Oldenburg, Germany; dana.schonvogel@dlr.de (D.S.); henrike.schmies@dlr.de (H.S.); p.wagner@dlr.de (P.W.); alexander.dyck@dlr.de (A.D.); 2 Institute of Chemistry, Carl von Ossietzky University, 26129 Oldenburg, Germany; frank.schroeter@uni-oldenburg.de (F.S.); michael.wark@uni-oldenburg.de (M.W.)

**Keywords:** nonprecious metal catalysts, Fe-N-C materials, activated biomass, porous carbon, carbon support, oxygen reduction reaction

## Abstract

Fe-N-C materials are promising non-precious metal catalysts for the oxygen reduction reaction in fuel cells and batteries. However, during the synthesis of these materials less active Fe-containing nanoparticles are formed in many cases which lead to a decrease in electrochemical activity and stability. In this study, we reveal the significant properties of the carbon support required for the successful incorporation of Fe-N-related active sites. The impact of two carbon blacks and two activated biomass-based carbons on the Fe-N-C synthesis is investigated and crucial support properties are identified. Carbon supports having low portions of amorphous carbon, moderate surface areas (>800 m^2^/g) and mesopores result in the successful incorporation of Fe and N on an atomic level and improved oxygen reduction reaction (ORR) activity. A low surface area and especially amorphous parts of the carbon promote the formation of metallic iron species covered by a graphitic layer. In contrast, highly microporous systems with amorphous carbon provoke the formation of less active iron carbides and carbon nanotubes. Overall, a phosphoric acid activated biomass is revealed as novel and sustainable carbon support for the formation of Fe-N_x_ sites. Overall, this study provides valuable and significant information for the future development of novel and sustainable carbon supports for Fe-N-C catalysts.

## 1. Introduction

The demand for energy and especially renewable energy sources is drastically increasing so that the conversion and storage of energy are the focus of current research. In particular, metal-air batteries and polymer electrolyte membrane fuel cell (PEMFC) systems become more and more prominent because of their ability to convert and store energy in an environmental friendly way. However, both systems are limited by the slow kinetics of the oxygen reduction reaction (ORR) that takes place on the cathode site [1,2]. Pt-based materials are used as appropriate catalysts, whereby the limited availability of Pt evokes high prices of raw material and can hinder the further commercialization of PEMFC-systems. Consequently, Pt-free of the so-called non-precious metal catalysts (NPMCs) are under intense research [2,3,4,5].

There are different types of NPMC like transition metal oxides, nitrides, or carbides, N- or B-doped carbons, or transition metal nitrogen species (Me-N_x_) incorporated into a graphitic carbon [6]. The latter material group, called Me-N-C, seems to be the most promising NPMC regarding stability and activity [2,4,5,6]. Among metallic sites consisting out of Co, Mn, Ni, or Cu, Fe in Me-N-C showed the best catalytic ORR performance in an acidic environment [5]. In general, the synthesis of these Fe-N-C materials is performed via pyrolysis of N-, metal-, and C-precursors at 700–1000 °C [3,4,7]. In order to produce such catalysts there are two different pathways, a template-based and a carbon support-based synthesis. Silica [8] or quite complex and expensive metal-organic-frameworks (MOFs) [9] can be used as a template, whereas for the support-based synthesis commercial carbon blacks [7,10] are utilized. The latter approach has the advantage of a simple and cheap synthesis without the requirement of a template removing step and the targeted use of carbon material with known morphology, porosity, surface area, and functionalities. These characteristics have a significant impact on the performance of fuel cell and battery electrodes [3]. However, one challenge of the Fe-N-C synthesis is the formation of unwanted nanoparticles during the pyrolysis, like metallic iron, iron carbide, iron nitride, or iron oxide which have low electrochemical ORR activity compared to carbon embedded Fe-N_x_ sites [11]. Kumar et al. reported that iron-containing particles also show moderate initial ORR activity in acidic electrolyte besides the atomic distributed Fe-N-C catalyst [12]. Other groups showed much lower activities for iron particles in acidic media compared to atomic Fe-N_x_ sites [13,14]. This is the reason why unprotected iron-containing particles are commonly removed by an additional step of acid leaching after pyrolysis [5]. However in many cases, iron particles are covered by a graphitic shell, making them inactive for the ORR on the one hand and hard to remove by leaching on the other hand. Choi et al. previously reported that protected particles that survived the acid leaching step dissolve at low potentials (<0.7 V vs. reversible hydrogen electrode (RHE)) during electrochemical experiments, promoting unwanted catalyst degradation. They assumed that the particles are not completely covered by graphite since graphitic layers can have nanometric defects or micropores and that due to the higher surface potential of the catalyst (open circuit potential of ~0.9 V vs. RHE) ferric hydroxides are formed which are stable during acid leaching but dissolve at lower potentials [15]. This is consistent with the low durability in acidic medium reported for Fe-N-C catalysts with metallic particles [12].

To avoid undesired iron particle formation during Fe-N-C catalyst synthesis, different parameters like iron content [16,17,18], reaction temperature [7], N- and Fe- precursors [19], or carbon properties [11] can be optimized. Especially since, in case of the support-based synthesis, the carbon material has a huge impact on atomic Fe-N_x_ site distribution and unwanted iron-containing particle formation. On one hand, a hierarchical carbon structure consisting of micro-, meso-, and macropores is most beneficial for heterogeneous catalysis, for example in fuel cell application [20]. On the other hand, micropores are discussed to be most important for the formation of active Fe-N_x_ sites [21,22,23]. Lefèvre et al. reported that when using a pore filler like cyanamide and a microporous carbon support, the active site formation proceeds mainly inside the micropores between two graphitic walls [21]. When ammonia was used as a N-source, a high amount of disordered carbon was shown to be beneficial besides the micropores [22,24]. On the contrary, Leonard et al. postulated that in addition, mesoporosity can have an impact on nitrogen incorporation and catalytic activity [25]. The influence of further physical properties of carbon support concerning undesired particle formation during the Fe-N-C synthesis was not reported in these studies. However, for the implementation of fuel cell and battery suitable carbon materials in Fe-N-C synthesis, the knowledge about the crucial carbon properties leading to an atomic distributed Fe-N-C catalyst is mandatory.

An important group of novel electrode materials are biomass-based activated carbons. On the one hand they provide sustainable material in contrast to crude-oil based carbon blacks, and on the other hand through precise chemical activation, micro- and mesoporosity in combination with a high surface area can be achieved [26,27]. For example, activation with KOH leads to microporous biochars, whereas activation with H_3_PO_4_ yield mesoporous carbon materials [28,29]. Furthermore, the initial composition of carbons and especially biochars can have a large influence on the suitability of these materials as electrode or catalyst support material, since transition metal traces and also metal complexes can be present [30].

In this study, crucial carbon support properties for the successful incorporation of atomic distributed Fe-N_x_ species are investigated and identified. Holistic physical analysis of four different carbon materials and their final Fe-N-Cs show that not only is the often-reported microporosity a critical factor for active site formation, but that also mesoporosity and amorphous carbon content play a role. Furthermore, the results will be verified by a comparison of electrochemical ORR activities. Two common carbon blacks, Vulcan^®^ and Black Pearls^®^, and two novel biomass-based activated carbons are used as supports. This study complements existing studies [21,22,25] regarding the influence of carbon supports on Fe-N-C synthesis by focusing on the origin of undesired metallic particle formation. Furthermore, we show the implementation of sustainable biomass-based carbons in Fe-N-C material and systematically investigate the effect of biomass activation process regarding Fe-N_x_-site incorporation and ORR activity.

## 2. Materials and Methods

### 2.1. Oxidation of Commercial Carbon Support

The oxidation of commercial carbon supports Black Pearls^®^ 2000 (BP) and Vulcan^®^ XC72R (V), both provided by Cabot (Boston, MA, USA), was carried out according to Schmies et al. [31]. A total of 2 g of carbon black was stirred in 200 mL of concentrated HNO_3_ (65 wt.%) purchased from Carl Roth (Karlsruhe, Germany) for 5 h at 90 °C, followed by filtration and washing with ultrapure water until neutral pH and drying in vacuum oven at room temperature for 24 h.

### 2.2. Activation of Biomass

Two processes were carried out to convert rye straw into activated carbon. The first process included the carbonization of rye straw inspired by the literature [28,32] with a heating rate of 5 °C/min to 400 °C and a holding time of 90 min under 100 L/h nitrogen flow in a ceramic tube furnace, yielding the biochar. In the second step, 1 g of the biochar was impregnated with KOH (≥85 wt.% p.a.), purchased from Carl Roth with a ratio of 1:3 and dried in a vacuum oven at room temperature for 12 h. The impregnated char was then transferred to a ceramic boat and put into a stainless steel tube furnace. Activation was carried out by heating to 720 °C at a heating rate of 5 °C/min and held for 1 h with 100 mL/min nitrogen flow, followed by intensive washing with 0.5 mol/L HCl (p.a.) from VWR (Radnor, PA, USA) and ultrapure water until a neutral pH, followed by drying in a vacuum oven at 30 °C. The KOH-activated pyrolyzed rye straw was denoted as aPRS_KOH_. The second activation process was adapted from Koyutürk et al. [29]. A total of 300 mg of rye straw was impregnated with H_3_PO_4_ (≥85 p.a.) purchased from Carl Roth with a biomass to acid ratio of 1:5. After drying for 48 h in a vacuum oven at 30 °C, the impregnated biomass was heated up to 580 °C at a heating rate of 5 °C/min and held for 3 h under a nitrogen flow of 100 mL/min in a stainless steel tube furnace. Afterwards the activated rye straw denoted as aRS_H3PO4_ was washed with 0.5 mol/L HCl and water until neutral pH and finally dried in vacuum oven at 150 °C.

### 2.3. Fe-N-C Synthesis

The synthesis of Fe-N-C was conducted according to the literature [7,18,33,34]. For the synthesis of Fe-N-C materials, 100 mg of carbon support was impregnated with a mixture of 16.25 mg iron(II) acetate (≥99.99%) and 421 mg of cyanamide (99%), both purchased from Sigma Aldrich (Darmstadt, Germany) in ethanol (Carl Roth) and mixed in a sonication bath until complete evaporation of the solvent and dried in vacuum oven at 30 °C overnight. Pyrolysis was carried out for 1 h at 900 °C at a heating rate of 5 °C/min and 100 L/h of nitrogen flow in a ceramic tube furnace followed by acid leaching with 2 mol/L of H_2_SO_4_ purchased from Carl Roth for 16 h at 90 °C. After washing the catalyst powder until neutral pH and drying, a second pyrolysis step similar to the first one was performed.

### 2.4. Methods

For scanning electron microscopy (SEM), a NEON 40 EsB CrossBeam instrument (Carl Zeiss, Oberkochen Germany) was used. Samples were fixed with adhesive carbon tape (Plano, Wetzlar, Germany) on a sample holder. For nitrogen sorption, the samples were evacuated at 150 °C overnight, and measurements were performed with TriStar II 3020 from Micromeritics (Unterschleißheim, Germany). Specific surface areas were calculated, applying the Brunauer–Emmett–Teller (BET) theory. Total pore volumes were determined at a relative pressure of 0.95, and micropore volumes were calculated using carbon black reference and the t-plot method [35,36]. Pore size distributions were acquired using the non-local density functional theory (NLDFT) carbon model of MicroActive software (version 3.0). The confocal Raman microscope Senterra from Bruker was used equipped with a He-Ne laser and light microscope BX51 (Olympus K.K., Tokyo, Japan). A laser power of 2 mW, 20× magnification, a wavelength of 633 nm, an integration time of 10 s, averaging of 10 scans and resolution of 3–5 cm^−1^ were used. Per sample, 3 different positions were measured and analyzed. Fitting of G- and D-bands was achieved using the software Unifit2020 (version 2020) by applying a linear background, four Lorentzian-shaped peaks, while applying a Gaussian-shaped peak for the D3-band according to Sadezky et al. [37]. Thermal gravimetric analysis (TGA) was carried out with TGA 4000 from Perkin Elmer (Rodgau, Germany) under nitrogen atmosphere using a flow rate of 40 mL/min. Transmission electron microscopy (TEM) images were taken with an EM902A system from Zeiss (Oberkochen, Germany) with 80 kV of acceleration voltage. The sample powder was dispersed in ethanol and deposited on a polyvinyl formal-coated copper grid with 200 meshes purchased from Plano. High resolution (HR) TEM images linked with energy dispersive X-ray spectroscopy (EDS) were recorded with a JEM-2100F from Jeol (Freising, Germany) with 80–200 kV accelerating voltage and INCA software (version 4.14) with 250 X-Max80 SDD detector from Oxford instruments (Abingdon, United Kingdom). Powder X-ray diffraction (XRD) measurements were acquired using the Empyrean series 2 instrument from PANalytical (Almelo, The Netherlands) in Bragg-Brentano-geometry using a Cu K_α_ radiation with 40 kV of voltage and a current of 40 mA. The software HighScore Plus (version 4.1) was utilized for data evaluation. The X-ray photoelectron spectra (XPS) were recorded at the ESCALAB 250Xi instrument by Thermo Fisher (Waltham, MA, USA) equipped with an Al K_α_ radiation. For survey spectra, 3 scans with a pass energy of 100 eV, dwell time of 20 ms, and step size 1 eV were averaged. For high resolution spectra, a pass energy of 20 eV, dwell time of 50 ms and step size 0.02 eV were chosen. Depending on the element, the numbers of scans were as follows: C1s 3 scans, O1s 5 scans, N1s 10 scans, and Fe2p 10 scans. No calibration of spectra was performed because no shift in graphitic carbon signal (284.4 eV) was observed. Spectra were fitted by the choice of a smart background and Gauss-Lorentz line shape and the Avantage software (version 5.9921) by Thermo Scientific. For the inductively coupled plasma mass spectrometry (ICP-MS), the XSeries2 device (Thermo Fisher Scientific) was used. Samples were prepared according to the literature by mixing 15 mg of catalyst with 2 mL of concentrated HNO_3_ (Rotipuran^®^Sup, Carl Roth) for 1 hat 100 °C [38]. After filtration, a solution of 50 mL using 2 vol% HNO_3_ was prepared followed by the addition of 1 mg/L of scandium as the internal standard. For Fe-N-aPRS_KOH_ and Fe-N-ox-V, a dilution by factor two was necessary. Calibration was done using Fe concentrations of 500, 1000, 2000, 3000, 4000, and 5000 µg/L (Carl Roth), ensuring a correlation coefficient of at least 0.999.

### 2.5. Electrochemical Measurement

For electrochemical characterization, a rotating disc electrode (RDE) setup was used including a glass cell with a standard hydrogen reference electrode (RHE), Pt-wire as counter electrode (CE), and 0.1 M HClO_4_ (Sigma Aldrich) as the electrolyte. The RHE and CE were separated by porous glass frites to exclude contaminations. Measurements were carried out using an Autolab potentiostat PGSTAT128N (Metrohm, Utrecht, The Netherlands). To achieve a catalyst loading of 400 µg/cm^2^, electrodes were coated with 12.6 µL of catalyst ink, consisting of catalyst powder (3 mg), ultrapure water (280.8 µL), 2-propanol (63 µL), and of a 5 wt.% Nafion solution (38.1 µL, Sigma Aldrich). The ink was dispersed by bath sonification for 30 min. For determination of ORR activity, the electrolyte was saturated with oxygen for 20 min followed by 3 cyclic voltammograms (CVs) in oxygen-saturated electrolyte at 1600 rpm in the range of 0.05–1.05 V_RHE_ with a scan rate of 5 mV/s. After the electrolyte was saturated with nitrogen for 20 min, the same CV procedure was used for capacitive current correction, followed by a determination of electrolyte resistance through electrochemical impedance spectroscopy in the range of 100 kHz–0.1 Hz at 0.3 V. Activity data were calculated from anodic scans and the onset potential corresponds to a current density of −0.1 mA/cm^2^. For the determination of mass activity, the kinetic current density j_kin_ was calculated by the Koutecky–Levich Equation (1), including the diffusion limited current density j_lim_ and current density j at 0.80 V_RHE_.

(1) $$j_{\mathrm{kin}}=\frac{j\cdot j_{\lim}}{j_{\lim}-j}$$

## 3. Results and Discussion

**Scheme 1:**
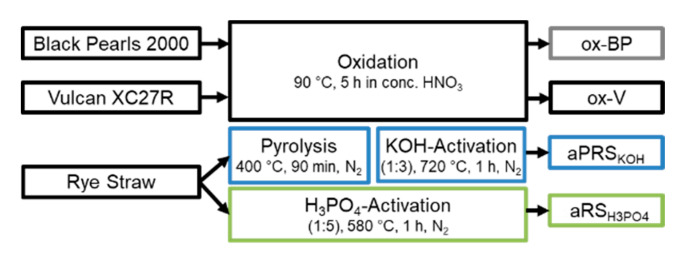
Schematic overview of material pre-treatment steps including experimental parameters and final notation of carbon supports.

Four different carbon materials were compared as support materials for the incorporation of Fe-N_x_-species into the graphitic network. Two commercial types of carbon black were used, namely Vulcan^®^ 72XCR and Black Pearls^®^ 2000. Both materials were oxidized previous to the catalyst synthesis with the aim of an improved incorporation of nitrogen following the usual procedure reported in the literature [7,33,39]. The oxidized carbon blacks are denoted as “ox-V” and “ox-BP”, as shown in Scheme 1. The XPS analysis of near-surface oxygen content revealed a molar fraction of 8.3 at.% for ox-V and 13.5 at.% for ox-BP (see Table S1). Furthermore, two biomass-based activated carbons obtained by activation of rye straw with H_3_PO_4_ or pyrolysis followed by KOH activation were investigated and are referred to aRS_H3PO4_ and aPRS_KOH_. An oxidation step for the biomass-based carbons was not necessary because the native oxygen contents were 11.3 at.% for aRS_H3PO4_ and 16.1 at.% for aPRS_KOH_ and thus comparable to oxidized commercial carbon blacks. For aRS_H3PO4_, there was also residual phosphor species of 4 at.% originating from the activation step detected in XPS measurement. Furthermore, nitrogen mainly attributed to pyridinic species was found for aPRS_KOH_ (5.9 at.%), aRS_H3PO4_ (2.5 at.%), and ox-BP (2.1 at.%). The N content in ox-BP originates from the residuals from the oxidation step [31], whereas the nitrogen of the biomasses can be attributed to natural nitrogen content [30].

### 3.1. Physical Properties of the Carbon Supports

**Figure 1:**
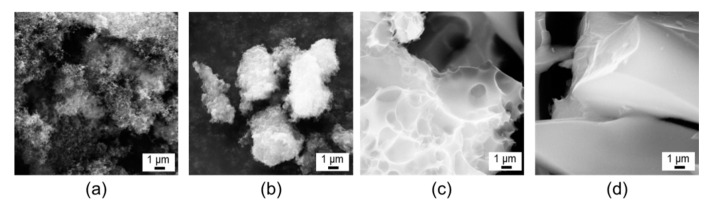
SEM images of (**a**) ox-V; (**b**) ox-BP; (**c**) aPRS_KOH_; and (**d**) aRS_H3PO4_.

The morphology of the four carbon supports was investigated by SEM as shown in Figure 1. For both, the ox-V as well as the ox-BP in Figure 1a,b small aggregated carbon particles (<100 nm) are observed as also seen in TEM images (see Figure S1). This was expected, as carbon blacks are typically formed through an oil furnace process leading to the formation of spherical aggregated particles [40]. On the other hand, the biomass-based carbons shown in Figure 1c,d contain much larger particles (≥100 nm) with a smoother surface in evidence. The aPRS_KOH_ shows the typical sponge-like structure which was observed by Shan et al. during the activation of bacterial cellulose as well [41]. Generally during KOH activation, K_2_CO_3_ is known to form and further decompose to CO_2_ and metallic K at a temperature higher than 700 °C [42,43]. Due to the CO_2_ formation during this chemical progress the formation of pores inside the carbon network takes place. Furthermore, the metallic K intercalates into the carbon matrix, leading to layer separation, which results in a micropore formation and high surface area [42,43,44]. On the other hand, the aRS_H3PO4_ in SEM indicates no macropores. The activation of biomass with phosphoric acid is according to Koyutürk et al., a sol-gel type carbonization process, where biomass is first dissolved in the acid followed by the formation of a porous carbon network [29].

**Figure 2:**
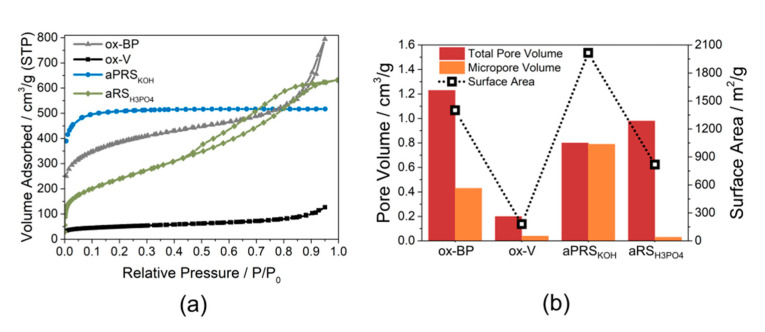
(**a**) Nitrogen sorption isotherms of four different carbon supports; (**b**) surface areas and total- and micropore volumes.

Nitrogen sorption experiments were carried out to investigate the porosity. The isotherms are shown in Figure 2a and can be classified according to IUPAC as follows: Type II for ox-V, type IVa for ox-BP, type Ia for aPRS_KOH_, and type IVa for aRS_H3PO4_ [45]. The type-II-isotherm is typical for less porous and furthermore macroporous carbons like Vulcan^®^ which is in agreement with the comparable low surface area of 179 m^2^/g and low pore volume of 0.2 cm^3^/g also shown in Figure 2b. The type-I-isotherm identifies microporous materials with a small external surface area and high micropore surface area [45]. This is the case for the KOH-activated biochar, which further shows a high specific surface area of 2015 m^2^/g and high micropore volume (Figure 2b). The type-IVa-isotherm is observed for mesoporous materials with pores larger than 4 nm and typically includes a hysteresis caused by pore condensation [45]. This is the case for aRS_H3PO4_ and ox-BP, whereby the latter material exhibits a hysteresis type H3, indicating the presence of macropores according to IUPAC [45]. For the aRS_H3PO4_ the hysteresis of the isotherm in Figure 2a already starts at low relative pressures of 0.4, indicating hysteresis type H4 and smaller mesopores compared to the ox-BP. Both materials show a comparable low micropore and high mesopore volume of 0.95 cm^3^/g in case of aRS_H3PO4_ and 0.80 cm^3^/g in case of ox-BP besides a moderate surface area higher than 800 m^2^/g in Figure 2b. Summarizing the N_2_ sorption results, three materials show a moderate surface area and porosity namely, ox-BP, aPRS_KOH_, and aRS_H3PO4_, whereas the ox-V has a low surface area and low pore volume.

**Figure 3:**
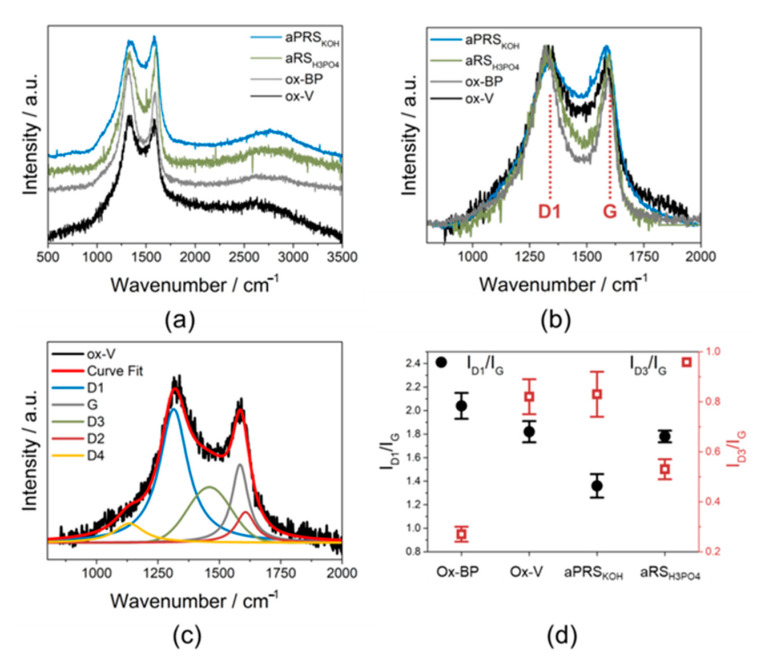
(**a**) Raman spectra of four different carbons; (**b**) first order Raman spectra with indicated D1- and G- band; (**c**) example of curve fitting of Raman spectra according to Sadezky et al. [37]; and (**d**) intensity ratios of D1/D3-band to G-band.

Raman spectroscopy was carried out to analyze the carbon structures and defective characters. In Figure 3a, the Raman spectra in range of 500–3500 cm^−1^ are shown, displaying the characteristic D- and G-bands for carbon materials. The first-order spectrum (800–2000 cm^−1^) is shown in Figure 3b. Typical for graphitic carbon materials, the D1-band (defect) and G-band (graphitic) at around 1320 cm^−1^ and 1590 cm^−1^ are observed. The intensities between the D1- and G-bands at around 1500 cm^−1^ are differing and indicate different amounts of amorphous carbon. Peak fitting according to Sadezky et al. [37] was carried out and is depicted in Figure 3c. Besides the well-known G-band which occurs for the ideal graphitic lattice, the D-band is split into four bands, namely D1─D4. D1, D2, and D4 represent disordered graphitic lattices. In detail, D1 (~1320 cm^−1^) is observed for graphitic edge planes, D2 (~1620 cm^−1^) for surface of graphitic layers, and D4 (~1350 cm^−1^) for polyenes and ionic impurities e.g., calcium, fluorine, or potassium. The D3-band (~1500 cm^−1^) can be found for amorphous and functionalized/heteroatom-doped carbons [5,37,46,47,48]. As no correlation between the amount of heteroatoms (O-,P-, and N-functionalities), which was higher for the biomass-based carbons compared to the carbon blacks (Supplementary Materials Table S1), and the D3-band was observed, the occurrence of the D3-band will be mostly attributed to amorphous carbon parts for interpretation. The determined peak height intensity ratios of the D1- to the G-band displayed in Figure 3d have values in range of 1.8–2.0, with the exception of aRS_KOH_ which shows a slightly lower value of around 1.4. As reported by Ferarri and Robertson, for highly graphitic materials a decrease in I_D1_/I_G_ indicates higher order whereas for carbons containing higher amounts of amorphous carbons (sp^3^) a decrease in I_D1_/I_G_ represents a higher disorder [49]. This is in agreement with the lower I_D1_/I_G_ ratio found for aPRS_KOH_ which was expected to be more amorphous due to the intercalation process during activation and resulting in high microporosity (Figure 2). The D3-/G-band intensity ratio analyses the extent of amorphous carbon [50] and illustrates distinct differences in the materials. For the aPRS_KOH_ as well as for the ox-V, significant higher values of around 0.8 compared to the aRS_H3PO4_ and ox-BP showing values lower than 0.5 were determined. Thus the latter two materials might have smaller parts of amorphous carbon. Low amounts of amorphous carbon and high parts of graphitized carbon are beneficial for the application as electrode material, however a too high degree of graphitization (I_D1_/I_G_ = 0.67) is reported to hinder the Fe-N_x_-site formation [51,52]. The discussed G and D bands can also be found as second–order bands in the in range of 2500–3000 cm^−1^ as a broad signal in Figure 3a. The broad signal can include the 2D overtone, G and D combination, as well as the 2D2 and 2D4 overtones which are typically found at 2700 cm^−1^, 2900 cm^−1^, 3100 cm^−1^, and 2400 cm^−1^ [37]. Due to low intensity, no peak fitting was carried out for the second-order region.

Summarizing the physical characterization, all four materials show a graphitic character making them suitable for fuel cell and battery electrodes [51]. Differences in morphology show larger C-particles for the biomass-based aPRS_KOH_ and aRS_H3PO4_. This might influence the final mass transport properties in electrodes but not the Fe-N-C synthesis, as both materials show moderate surface areas above 800 m^2^/g and high pore volumes that are reported to be beneficial for Fe-N-C synthesis [2]. This is also the case for ox-BP, whereas the ox-V, which is often used as support for Pt-nanoparticles, shows low surface area and pore volume which might hinder Fe-N_x_ site incorporation. Furthermore, differences in the portion of amorphous carbon were observed, being slightly higher for the ox-V and aPRS_KOH_ materials.

### 3.2. Application of Carbon Supports in Fe-N-C Synthesis

**Figure 4:**
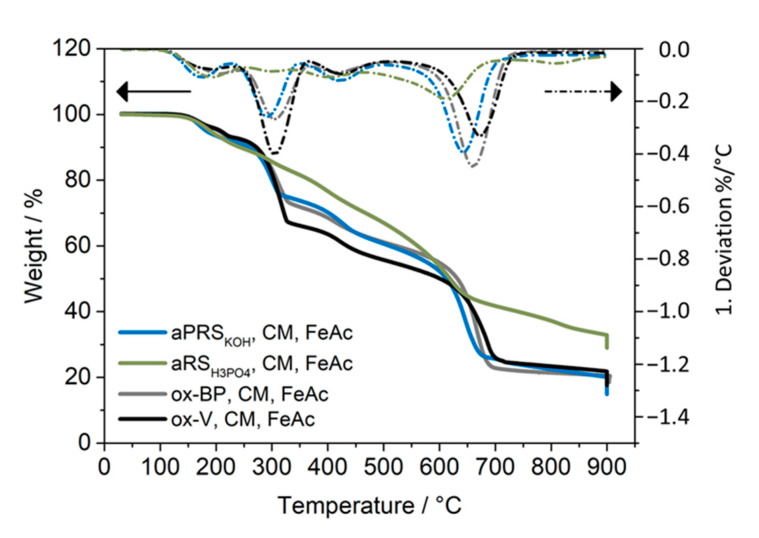
Thermogravimetric analysis with mass loss and first deviation during synthesis of Fe-N-aPRS_KOH_, Fe-N-aRS_H3PO4_. Fe-N-ox-BP and Fe-N-ox-V, using cyanamide (CM) and iron(II) acetate (FeAc).

Synthesis of Fe-N-C proceeded as commonly reported in the literature including the impregnation of supports with cyanamide and iron(II) acetate and first pyrolysis for 1 h at 900 °C under N_2_ atmosphere, followed by acid leaching with sulphuric acid and a second equal pyrolysis step [7,34]. To gain information about formation and decomposition steps during Fe-N-C synthesis, the mass loss of the first pyrolysis step was followed by thermogravimetric analysis. Thermograms of the impregnated carbon supports (18.6 wt.%) containing 3 wt.% iron acetate and 78.4 wt.% cyanamide as well as their first derivative are displayed in Figure 4. Thermogravimetric curves in Figure 4 prove that cyanamide condenses to melamine up to 350 °C followed by the partial sublimation of melamine. At around 520 °C, graphitic carbon nitrides are formed and then decompose at ~600 °C to form graphitic carbon and nitrogen-containing gases like ammonia and cyano fragments. These nitrogen-containing species promote nitrogen incorporation [53,54]. Iron(II)acetate starts to decompose at temperatures of around 300 °C, forming iron oxide species andiron carbide at elevated temperatures [14,55]. Li et al. recently found, by using in-temperature X-ray absorption spectroscopy, that at temperatures in range of 600–1000 °C the iron oxide species can be transformed to atomic Fe species. They can in turn coordinate to the nitrogen species that are incorporated in the carbon network and can form Fe-N_x_ sites [14].

In case of ox-BP, ox-V, and aPRS_KOH_ four mass loss steps with maximal loss at around 180, 300, 420, and 650 °C can be observed. The two first mass losses can be assigned to the decomposition of oxygen functionalities and sublimation of formed melamine. The other two mass losses at 420 °C and 650 °C are related to the formation of graphitic carbon nitrides and their decomposition into ammonia containing gases at elevated temperatures. aRS_H3PO4_ shows less distinct mass losses and an additional mass loss step at 820 °C in Figure 4, that might origin from the degradation of instable carbons. In particular, the distinct mass loss at around 300 °C is missing for aRS_H3PO4_, whereas the mass loss step assigned to decomposition of graphitic carbon nitrides is broader and shifted to a lower temperature of 615 °C compared to the other materials. This indicates a different process of cyanamide decomposition and nitrogen incorporation which might be caused by the phosphorus species in aRS_H3PO4_. Furthermore, the mass loss after heat treatment in TGA counts 71 wt.% in case of aRS_H3PO4_ and is lower compared to the other materials that show mass losses in the range of 82–86 wt.%. A repetition of measurements with a fresh sample yielded the same thermogram. The weights measured before and after the first pyrolysis of synthesis in a tube furnace show comparable mass losses for all supports in the range of 80–82 wt.%, that are comparable to mass losses reported by Tian et al. [18]. Moreover, the sublimation of melamine was observed for all four samples, as small resublimed amounts were observed on the furnace exit. Differences between TGA measurement and real synthesis in a tube furnace might be due to different scales or rather weights used on the one hand or an influence of the above mentioned phosphorus species in aRS_H3PO4_ on the other hand.

### 3.3. Impact of Carbon Support on Fe-N_x_ Site Incorporation

**Figure 5:**
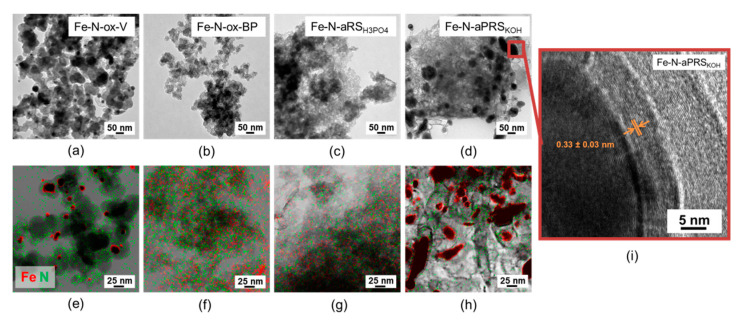
(**a**–**d**) TEM images; (**e**–**h**) High Resolution-TEM/EDS mapping of Fe and N of the four Fe-N-C materials, and (**i**) HR-TEM image with indicated lattice distance of Fe-N-aRS_KOH_.

Finally, the synthesized Fe-N-C materials were characterized concerning their physical properties using TEM, High resolutionTEM/EDS, XRD, XPS, and N_2_ sorption. Figure 5 shows TEM images of the four Fe-N-C samples as well as HR-TEM images with EDS mappings of the elements iron and nitrogen. The mapping of C was excluded because the TEM grid polyvinyl formal film also contains C which is shown in the Supporting information (Supplementary Materials Figure S2).

**Figure 6:**
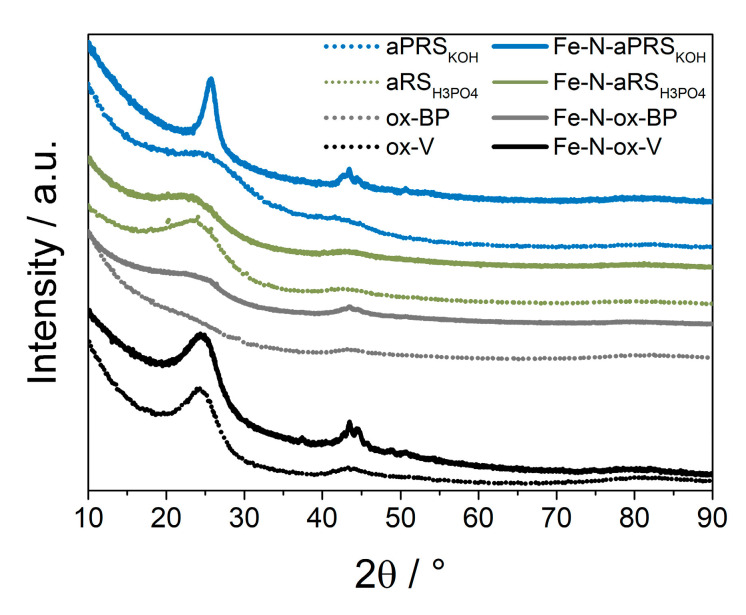
XRD of neat carbon supports as well as final Fe-N-C materials.

For the ox-V and aPRS_KOH_-based Fe-N-C materials, highly visible and undesired metallic particle formation has been observed. A closer look using HR-TEM and EDS in Figure 5e,h,i revealed that the particles on both materials consist of iron and are covered by a graphitic shell, which obviously hindered their acid leaching. The graphitic carbon was identified by the lattice distance of 0.33 ± 0.03 nm which is referred to as the 002 crystal lattice distance of graphite (0.3324 nm according to ICDD 98-018-7640). Figure 5e,h further show the absence of distributed single Fe atom sites in Fe-N-ox-V and Fe-N-aPRS_KOH_. However, the nitrogen doping of carbon seems not to be affected by the presence of Fe-containing particles since nitrogen atoms are uniformly distributed on the carbon surfaces of both materials. Furthermore, the KOH-activated Fe-N-C in Figure 5d shows the presence of bamboo-like nanotubes. The formation of carbon nanotubes is catalyzed by iron species present on the carbon surface and was observed by Strickland et al. as well [56]. On the other hand, for the Fe-N-ox-V sample no nanotubes in Figure 5a can be seen. Since Fe-N-ox-V also contains strongly visible Fe-specified particles, the absence of carbon nanotube formation indicates a different nature of iron-containing particles here, like metallic iron or Fe_3_C. With a look at the TEM images of the ox-BP and aRS_H3PO4_-based Fe-N-C materials in Figure 5f,g comparable metallic particles on the carbon surface cannot be observed. Instead, a uniform incorporation of Fe and N is verified by the EDS mapping. At this point, we can state that two supports failed to form an atomic distributed Fe-N-C catalyst, namely ox-V and aPRS_KOH_, and two supports lead to an applicable Fe-N-C catalyst. This observation is further supported by the X-ray diffractograms shown in Figure 6.

**Figure 7:**
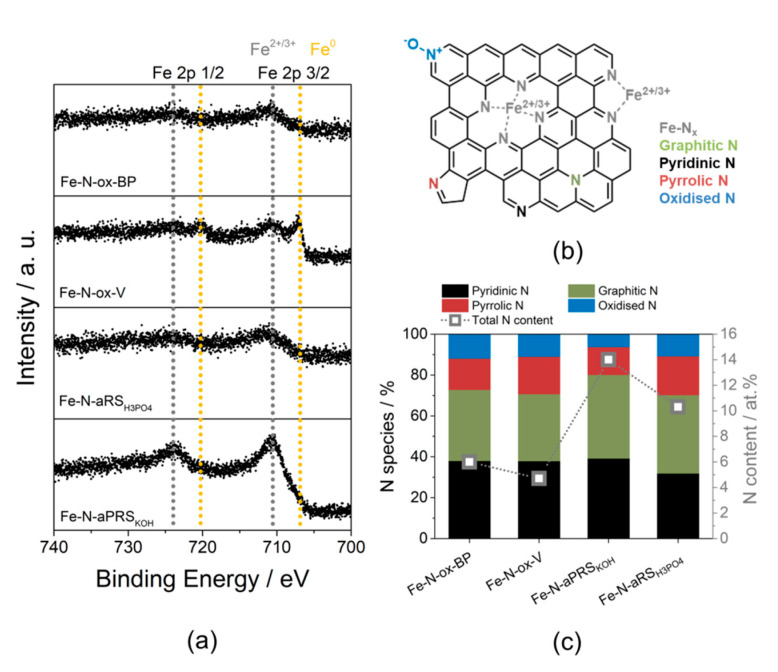
(**a**) Fe 2p XP-spectra of Fe-N-C materials; (**b**) the proposed structure of Fe-N-C material; and (**c**) ratios of nitrogen functionalities and total nitrogen content.

From XRD, a comparison of the neat carbons with the final Fe-N-C samples in Figure 6 shows no distinct changes after Fe-N-C synthesis for the ox-BP (marked grey) and aRS_H3PO4_ (marked green), where mainly the typical broad graphitic signals at ~25° (002) and ~44° (011) can be seen (ICDD 98-061-7290) assigned to amorphous carbon with small graphitic crystallites [12], as also observed in the Raman spectra of the supports (Figure 3). The absence of sharp peaks indicates an absence of crystalline iron-containing particles. However, the diffractograms of Fe-N-ox-V (marked black) and Fe-N-aPRS_KOH_ (marked blue) have low intensity but sharp peaks beside the carbon peaks in the range of 40–50°. Those cannot be properly identified due to small intensity but are presumed to be assigned to metallic Fe or Fe_3_C as reported in the literature [12,57,58]. Furthermore, the C (002) reflection becomes sharper for the Fe-N-aPRS_KOH_ sample. The increase in sharpness is due to the iron compound (e.g., Fe_3_C) induced graphitization and formation of graphitic nanotubes [58]. This indicates again a different interaction of Fe with C, because no increase of graphitic peak and nanotube formation is observed for the ox-V after Fe-N-C synthesis. To determine the bulk iron content in the samples, ICP-MS measurements were carried out. Fe-N-ox-BP as well as the Fe-N-aRS_H3PO4_ samples reveal comparable Fe amounts of 1.33 wt.% and 1.20 wt.%, whereas the Fe-N-aPRS_KOH_ showed a slightly higher content of 1.87 wt.% (Supplementary Materials Table S2). A sample with pure BP resulted in a low Fe content of 0.04 wt.%, which is caused by possible iron traces in the sample and used chemicals itself on one hand and possible interferences in the mass spectrometer on other hand. However, for the Fe-N-ox-V sample, a significantly higher amount of 2.69 wt.% was found. The higher Fe contents of the Fe-N-ox-V and Fe-N-aPRS_KOH_ sample are attributed to the formation of large iron carbide and iron particles, as already observed in EDS (Figure 5) and XRD (Figure 6). The near-surface elemental composition was determined by XPS. For all four materials, the presence of C (78–91 at.%), O (2–9 at.%), N (6–14 at.%), and Fe (0.4–1.1 at.%) was observed (see Supplementary Materials Table S2). For the Fe-N-aRS_H3PO4_ material 2 at.% of P was also determined to originate from the activation step. The oxygen content after Fe-N-C synthesis decreases in the range of 7–11 at.% (see Supplementary Materials Tables S1 and S2), which was expected as oxygen functionalities decomposes during synthesis to enable nitrogen incorporation [39]. Due to the expected and typical small amount of iron ≤0.4 at.% in the Fe-N-C materials, peak fitting was not carried out for the Fe2p XP-spectra in Figure 7a. Nevertheless, all Fe-N-C materials show a broad signal at 710.6 eV that can be assigned to the Fe2p 3/2 signal of Fe^2+^/Fe^3+^ as also observed for Fe-N-C materials in other studies [4,59,60] and indicates the Fe-N_x_ sites or iron carbide. Fe-N-ox-V has, contrary to the others, a further Fe2p 3/2 peak at 706.9 eV and the related Fe2p 1/2 peak at 720.0 eV which is known for Fe^0^ and verifies the presence of metallic iron which is also in agreement with TEM results (Figure 5) [14]. Therefore, it is assumed that in case of Fe-N-aPRS_KOH_ mainly Fe_3_C is formed, whereas Fe-N-ox-V consists of Fe as well as Fe_3_C particles. The presence of Fe_3_C in larger parts might explain the results of TEM and XRD where for Fe-N-aPRS_KOH_ carbon nanotube formation was observed, whereas no nanotubes have been seen in TEM for the Fe-N-ox-V.

**Figure 8:**
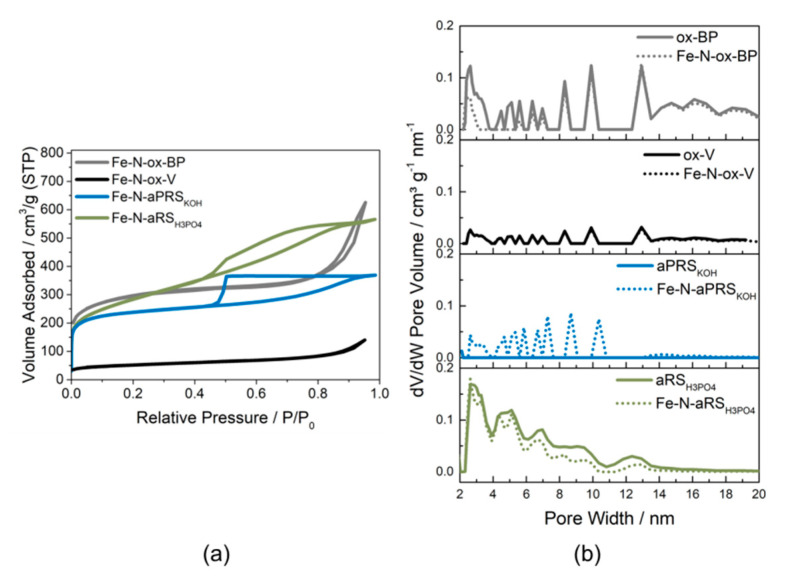
(**a**) Nitrogen sorption isotherms of Fe-N-C materials and (**b**) pore size distribution of carbon supports and Fe-N-C materials.

Four different N-species can be obtained by deconvolution of the N1s spectra (see Supplementary Materials Figure S3), namely pyridinic (396.3 eV), pyrrolic (400.0 eV), graphitic (400.8 eV), and oxidized nitrogen (403.6 eV), as schematically shown in Figure 7b. All N-species are incorporated into the graphitic carbon network and pyridinic N can further coordinate with Fe to form Fe-N_x_ sites [17]. Peak fitting was performed according to Hu et al. [4] and total N-content, as well as ratios of the four different N-species, is presented in Figure 7c. Both commercial carbon-based Fe-N-C samples show a nitrogen content of around 5 at.%, whereas significant higher nitrogen amounts with more than 10 at.% for the biomass-based Fe-N-C sample were achieved. On the one hand, this is assigned to the native nitrogen content of the biomass-based materials being 2.5–5.9 at.% (Supplementary Materials Table S1). On the one hand, the higher amount of carbonyl functionalities in aPRS_KOH_ and aRS_H3PO4_ that decompose during Fe-N-C synthesis forming defect sites, seem to be beneficial for increased nitrogen incorporation (Supplementary Materials Table S1). Looking at the ratios of the N-species, all Fe-N-C materials show mainly graphitic and pyridinic N groups as found for other Fe-N-C in literature as well [4,61]. No significant differences between the four materials are observed which would lead to a clear indication as to which N-species would be favorable for undesired particle formation. This is in accordance with the well-distributed nitrogen in EDS mapping in Figure 5 and verifies that Fe-containing particle formation does not affect the nitrogen doping itself. Nitrogen doping obviously takes place before the Fe-conversion during synthesis as already suggested in the previously shown TGA curves in Figure 4. Li et al. recently demonstrated that the iron precursor first forms iron oxide Fe-O_6_ (octahedral) followed by the conversion to Fe(II)-O_4_ (tetrahedral). This is then converted to atomic Fe and finally, at temperatures of around 600 °C the coordination to doped N happens, forming active Fe(II)-N_4_ sites [14]. At a temperature above 600 °C, the Fe(II)-O_4_ and Fe-N_4_ site formation compete. Fe-N_4_ sites are more thermally stable and therefore predominant if appropriate amounts of nitrogen doping are present. It was also shown that if no appropriate nitrogen doping exists, the iron remains as oxide or forms Fe_x_ metal cluster [14]. According to that study, the achieved two times larger N-content of the biomass-based carbons in Figure 7c seems in principle beneficial for iron atom incorporation in Fe-N-C synthesis. Interestingly, aPRS_KOH_ shows that despite the large nitrogen doping, a distinct Fe_3_C particle formation can be seen in Figure 5 and Figure 7a. Therefore, further impact factors must play a significant role in synthesis. Thus, further detailed analysis towards porosity and pore sizes was performed. Figure 8 shows the sorption isotherms of Fe-N-C materials and pore size distributions in the range of 2–20 nm.

The Fe-N-C samples based on ox-BP and aRS_H3PO4_, which turned out as atomic distributed Fe-N-C material, both retain the type IV isotherm and their hysteresis after Fe-N_x_ incorporation (cf. Figure 2). Furthermore, the pore size distributions in Figure 8b, show for both materials a lower amount of mesopores after Fe-N-C synthesis, which are predominant in the range of 2–6 nm for ox-BP and 2–14 nm for aRS_H3PO4_. This demonstrates that a part of the active site formation impacts the small mesopores. The Fe-N_x_ site formation in mesopores was also assumed by Leonard et al. in a previous study but not further presumed to prevent undesired iron containing particle formation [25]. With a look at the pore size distribution of Fe-N-ox-V in Figure 8b, which showed metallic particle formation during synthesis, nearly no change in mesopore distribution between native carbon and Fe-N-ox-Vulcan can be observed. This supports the hypothesis that the active sites are formed in small mesopores. Moreover, the native ox-V already showed a low pore volume of 0.2 cm^3^/g and low amount of mesopores in Table 1, which can be the reason for predominant particle formation during Fe-N-C synthesis. If the porosity and surface area are too small, the carbon surface is less accessible and the precursors cyanamide and iron(II) acetate cannot be properly distributed to ensure formation of the desired surface sites.

On the other hand, the carbon support aPRS_KOH_ showed the largest specific surface area of 2015 m^2^/g in Table 1 but a morphology restricted to micropores. Since the carbon surface is for this reason less accessible to the Fe-/N-precursors, less Fe-N_x_ sites and instead other Fe-containing particles are formed. Indeed, the final Fe-N-aPRS_KOH_ shows unwanted Fe_3_C particles and furthermore, carbon nanotubes. Figure 8b shows generated mesopores in Fe-N-aPRS_KOH_ after synthesis, which are assigned to these significant structural changes in terms of Fe_3_C particle and carbon nanotube formation. We conclude at this point that a sufficiently high specific surface area, exceeding the one of ox-V of around 179 m^2^/g, and moreover the presence of mesopores are essential in Fe-N-C synthesis. Furthermore, different pore structures of ox-V and aPRS_KOH_ could be the cause for the different Fe-particle species in both materials. Possibly the Vulcan^®^ support is not able to interact with the iron due to low pore volume of and also small surface area (see Table 1) so that metallic iron particles instead of iron carbides are formed. These particles become covered by a graphitic shell originating from cyanamide, which is present in excess on the surface due to limited porosity. At the same time aPRS_KOH_ might adsorb more cyanamide and iron(II) acetate, presumably more homogeneously as well, due to a higher surface area and more micropores that can be filled. However, the micropores obviously collapse after Fe-N-C synthesis, as evidenced by the decrease of SSA by 57% and micropore volume by 65%, leading to iron carbide formation. These Fe_3_C particles can further promote the formation of CNTs as reported in the literature and verified in this study by TEM analysis in Figure 5d [58,62]. The collapse of micropores and formation of nanotubes lead then to the generation of the mesopores observed in the pore size distribution of Figure 8b.

**Figure 9:**
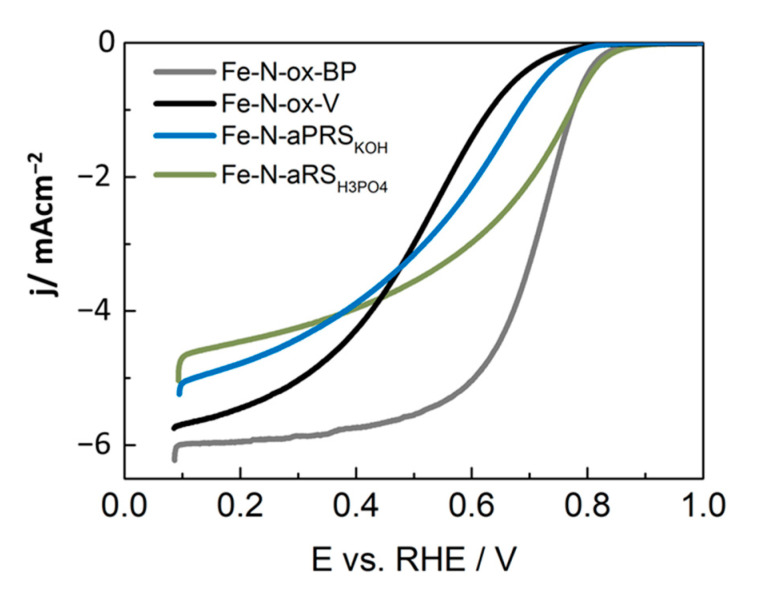
Linear sweep voltamogramms of the four Fe-N-C catalysts with a loading of 400 µg/cm^2^ at a rotation speed of 1600 rpm in O_2_-saturated 0.1 M HClO_4_ electrolyte.

Finally, the Fe-N-C materials were applied as catalysts for the ORR in acidic media to verify the results of physical analysis and point out the significant influence of different active sites e.g., Fe-N_x_, Fe_3_C, and Fe particles towards the ORR activity. Figure 9 displays the linear sweep voltammogram for each catalyst in 0.1 M HClO_4_ electrolyte, showing differences in the diffusion- (<0.4 V_RHE_) as well as kinetic-limited (>0.7 V_RHE_) regions. The lower negative current densities in the diffusion-limited region appearing for the biomass-based catalysts can be due to the larger particle sizes of biomass (Figure 1). For a comparison of ORR activity, onset potentials and mass activities at 0.80 V_RHE_ were determined. The Fe-N-ox-BP and Fe-N-aRS_H3PO4_ catalyst show the highest onset potentials with values of 0.844 V_RHE_ and 0.857 V_RHE_, verifying the presence of high ORR active Fe-N_x_ sites. The Fe-N-aPRS_KOH_ and Fe-N-ox-V catalysts containing larger iron and iron carbide particles, on the other hand, have significantly lower onset potentials of 0.789 V_RHE_ and 0.769 V_RHE_ that indicate lower ORR activity. The determined mass activities at 0.80 V_RHE_ are 1.27 A/g and 1.52 A/g for Fe-N-ox-BP and Fe-N-aRS_H3PO4_, being highly comparable to activities reported for Fe-N-C catalysts in the literature [25,38]. Fe-N-aRS_KOH_ and Fe-N-ox-V have significant lower mass activities with values of 0.19 A/g and 0.13 A/g. The lower onset potentials and mass activities are in agreement with the results of physical analysis, showing mainly Fe_3_C for the Fe-N-aPRS_KOH_ catalyst and metallic Fe for the Fe-N-ox-V catalyst, which are known to be less active towards the ORR in acidic electrolyte [13]. These differences in mass activity further demonstrate that the selection of carbon support is essential as the formation of iron carbide and metallic iron species leads to a noticeable deterioration of ORR activity.

In summary, the relevant properties of the carbon support for atomic Fe-N_x_ site incorporation and high ORR activity are listed in Table 2 on the basis of the presented physical and electrochemical characterization. The carbon supports, having low amorphous carbon contents, moderate surface areas (>800 m^2^/g), and mesoporosity like in Black Pearls^®^ and phosphoric acid activated rye straw (aRS_H3PO4_), result in successful Fe-N-C synthesis with atomically distributed Fe and N and enhanced ORR performance. On the other hand, by using low surface area carbon with a higher amorphous character like Vulcan^®^ in Fe-N-C synthesis, mainly metallic iron species covered by a graphitic layer are formed, leading to a less active ORR catalyst. The application of a highly microporous system with amorphous carbon e.g., KOH-activated biomass (aPRS_KOH_) provokes the formation of iron carbides, which catalyze carbon nanotube generation going along with a collapse of the original carbon network and low ORR performance.

## 4. Conclusions

Significant properties of carbon support materials on the successful synthesis of ORR active Fe-N-C catalysts have been investigated. Two commonly available carbon blacks (Black Pearls^®^ and Vulcan^®^) as well as two novel activated biochars based on rye straw were characterized concerning their physical properties like carbon structure and porosity. The four carbon supports were applied in Fe-N-C synthesis and analyzed regarding their incorporation of N- and Fe-atoms as well as their activity towards ORR in acidic electrolyte. We conclude that carbon supports with low amorphous parts, a surface area higher than 800 m^2^/g, and the presence of mesopores are required for incorporation of mainly atomic Fe-N sites and thus high ORR activities. This study helps to push the development of optimized carbon supports in Fe-N-C ORR catalysts forward, which are important in renewable energy systems like PEMFCs. Furthermore, we implemented H_3_PO_4_-activated rye straw as novel carbon support in Fe-N-C synthesis and showed its promising ORR activity, which is significant for the further development of more sustainable ORR catalysts.

## Figures and Tables

**Table 1:** Specific surface area (SSA), pore volume (PV), micropore volume (MiPV), and micropore area (MiA) derived from the N_2_ sorption experiment data.

Sample	SSA/m^2^/g	PV/cm^3^/g	MiPV/cm^3^/g	MiA m^2^/g
ox-BP	1400	1.23	0.43	991
Fe-N-ox-BP	1280	1.19	0.39	902
ox-V	179	0.20	0.04	89
Fe-N-ox-V	172	0.22	0.04	95
aPRS_KOH_	2015	0.80	0.79	1998
Fe-N-aPRS_KOH_	863	0.56	0.28	675
aRS_H3PO4_	818	0.98	0.03	37
Fe-N-aRS_H3PO4_	977	0.88	0.11	214

**Table 2:** Summary of physical properties of four different carbon supports influencing Fe-N_x_ site incorporation, including results of morphology analyzed by SEM, specific surface area as well as porosity determined by N_2_ sorption and carbon structure evaluated by Raman spectroscopy.

Parameter	Ox-BP	Ox-V	aPRS_KOH_	aRS_H3PO4_
Morphology (SEM, Figure 1)	Small C-particles (<100 nm)	Small C-particles (<100 nm)	Large C-particles (≥100 nm)	Large C-particles (≥100 nm)
Surface area (N_2_ sorption, Figure 2)	Moderate SA (≥800 m^2^/g)	Low SA (<800 m^^2^^/g)	Moderate SA (≥800 m^2^/g)	Moderate SA (≥800 m^2^/g)
Pore volume (N_2_ sorption, Table 1)	Moderate PV (≥0.8 cm^3^/g)	Low PV (<0.8 cm^3^/g)	Moderate PV (≥0.8 cm^3^/g)	Moderate PV (≥0.8 cm^3^/g)
Porosity (N_2_ sorption, Table 1)	Micro + Meso	-	Micro	Meso
Carbon structure (Raman, Figure 3)	Less amorphous (I_D3_/I_G_ < 0.8)	More amorphous (I_D3_/I_G_ ≥ 0.8)	More amorphous (I_D3_/I_G_ ≥ 0.8)	Less amorphous (I_D3_/I_G_ < 0.8)
Incorporation of Fe-N_x_-sites	Successful	Mainly Fe particle	Mainly Fe_3_C particle	Successful
ORR activity of Fe-N-C@ 0.80 V_RHE_ (LSV, Figure 9)	Improved (1.27 A/g)	Low (0.13 A/g)	Low (0.19 A/g)	Improved (1.52 A/g)

## Data Availability

The data presented in this study are available on request from the corresponding author.

## Supplementary Materials

The following are available online at https://www.mdpi.com/1996-1944/14/1/45/s1, Table S1: Near-surface elemental content of KOH- and H_3_PO_3_ activated rye straw (RS) and oxidized Vulcan^®^ and Black Pearls^®^ in at.% as determined from XPS analysis, Table S2: Near-surface elemental content of Fe-N-C materials in at.% as determined from XPS analysis and Fe bulk content determined via ICP-MS measurements in wt.%, Figure S1: TEM images of (a) ox-V; (b) ox-BP; (c) aPRS_KOH_; (d) aRS_H3PO4_, Figure S2: TEM/EDS mapping of the four Fe-N-C materials, Figure S3: XP-spectra of Fe-N-C catalysts, (a) C1s, C=C (284.4 eV), C-O/C-N (285.7 eV), C=O (287.1 eV) satellite (289.9 eV), artefact (283.8 eV); (b) N1s, pyridinic (396.3 eV), pyrrolic (400.0 eV), graphitic (400.8 eV), oxidized nitrogen (403.6 eV); (c) O1s, C-O (532.9 eV), C=O(531.2 eV), satellite (537.3 eV).
