# Supplementary material for: Relevant Properties of Carbon Support Materials in Successful Fe-N-C Synthesis for the Oxygen Reduction Reaction: Study of Carbon Blacks and Biomass-Based Carbons
Source: Materials (Basel). 2020 Dec 24;14(1):45. doi: 10.3390/ma14010045 (PMC7795179; doi:10.3390/ma14010045)
Supplement: Supplementary file 1 [file materials-14-00045-s001.pdf]

materials

Supporting Information

Relevant Properties of Carbon Support Materials in Successful Fe-N-C Synthesis for the Oxygen Reduction Reaction: Study of Carbon Blacks and Biomass-Based Carbons

Julia Hülstede, Dana Schonvogel, Henrike Schmies, Peter Wagner, Frank Schröter, Alexander Dyck and Michael Wark

Table S1: Near-surface elemental content of KOH- and H_3PO_3 activated rye straw (RS) and oxidized Vulcan® and Black Pearls® in at% as determined from XPS analysis.

		**Ox-BP**	**Ox-V**	**$aPRS_{KOH}$**	**aRS_{H3PO4}**
C1s	C=C	70.4	80.3	60.5	61.5
	C-O	9.6	8.2	9.0	11.7
	C=O	4.5	3.2	8.5	3.4
O1s	C-O	9.9	5.0	8.3	6.3
	C=O	3.6	3.3	7.8	5.0
	P-O-P	-	-	-	5.5
N1s	$N_{Pyr, -NH2, -NH}$	1.5	-	5.9	2.5
	N_{NO3}	0.6	-	-	-
P2p	P_x	-	-	-	4.1

Table S2: Near-surface elemental content of Fe-N-C materials in at% as determined from XPS analysis and Fe bulk content determined via ICP-MS measurements in wt%

		**Fe-N-ox-BP**	**Fe-N-ox-V**	**Fe-N-$aPRS_{KOH}$**	**Fe-N-aRS_{H3PO4}**
C1s / at%	C=C	74.5	78.9	63.8	58.6
	C-O C-N	11.4	9.75	10.5	12.5
	C=O	5.3	4.5	4.7	7.13
O1s / at%	C-O	1.0	0.83	2.8	3.57
	C=O	1.4	0.85	3.1	3.51
	P-O-P	-	-	-	1.9
N1 / at%	Pyridinic N	2.3	1.8	5.5	3.3
	Pyrrolic N	0.9	0.9	1.9	2.0
	Graphitic N	2.1	1.5	5.7	3.9

	Oxidised N	0.7	0.5	0.9	1.1
$P2p_{3/2}$ / at%	P-x	-	-	-	2.2
$Fe2p_{3/2}$*/ at%	Fe^{2+}/Fe^{3+}	0.4	0.18	0	0.4
	Fe^{0}	0	0.26	1.1	-
Fe /wt%	ICP-MS	1.33	2.69	1.87	1.20

*Values only used as rough guide value, as peak fitting showed relative high fitting deviation (residual)

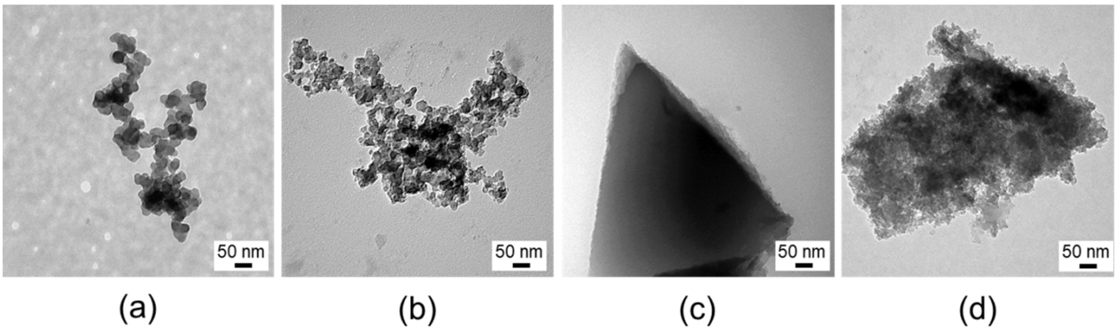

Figure S1: TEM images of (a) ox-V; (b) ox-BP; (c) $aPRS_{KOH}$; (d) aRS_{H3PO4}.

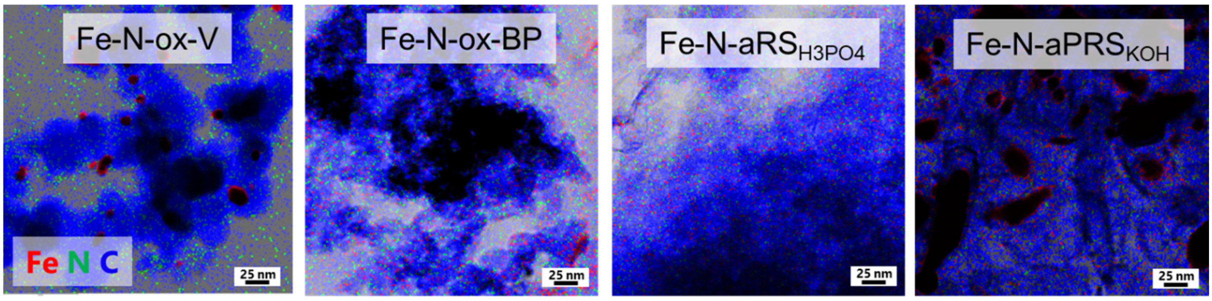

Figure S2: TEM/EDS mapping of the four Fe-N-C materials.

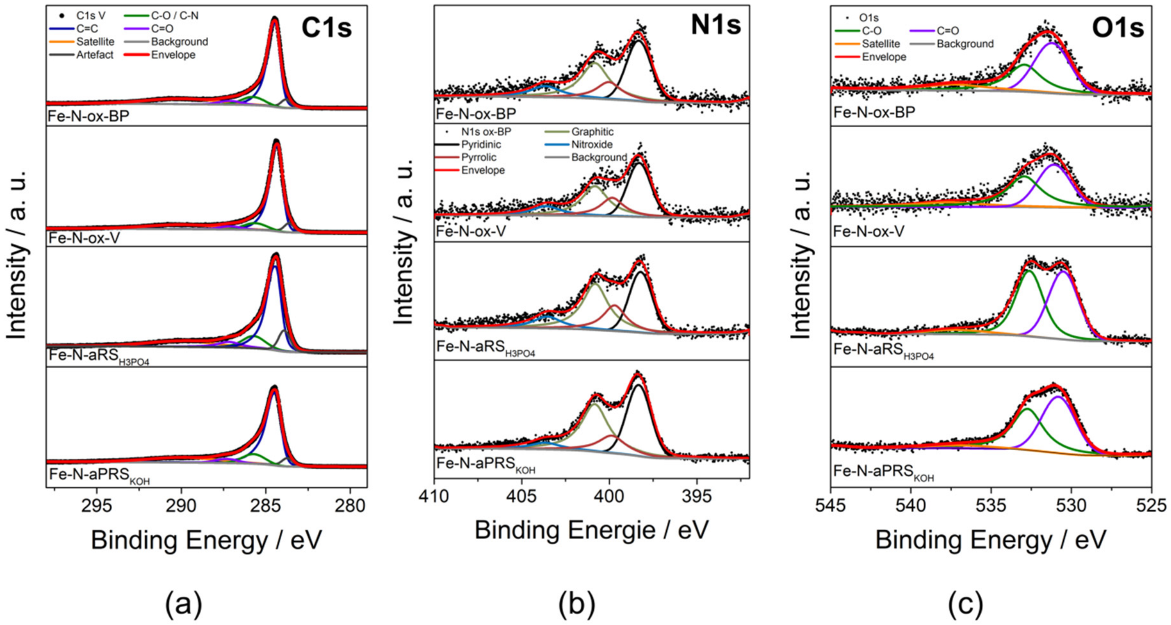

Figure S3: XP-spectra of Fe-N-C catalysts, (a) C1s, C=C (284.4 eV), C-O/C-N (285.7 eV), C=O (287.1 eV) satellite (289.9 eV), artefact (283.8 eV); (b) N1s, pyridinic (396.3 eV), pyrrolic (400.0 eV), graphitic (400.8 eV), oxidized nitrogen (403.6 eV); (c) O1s, C-O (532.9 eV), C=O(531.2 eV), satellite (537.3 eV).
